# Colour-coded density-gradients stone mapping: A novel reporting system for stone density on non-contrast computed tomography and its clinical applications

**DOI:** 10.1080/2090598X.2020.1784601

**Published:** 2020-07-07

**Authors:** Mohamed Adel Atta, Hussein Mamdouh Abdeldaeim, Mohamed Mohie Eldin Hashad, Mohamed Samir Shabaan

**Affiliations:** Faculty of Medicine, Alexandria University, Alexandria, Egypt

**Keywords:** Non-contrast CT, colour coding, density gradients, stones

## Abstract

**Objective:**

To presents a novel clinically oriented system to report stone attenuation on non-contrast computed tomography (NCCT) using colour-coded density-gradients stone mapping and its clinical applications.

**Patients and methods:**

This study was performed on 50 patients with 63 stones. All patients had a recent history of failed shockwave lithotripsy (SWL) or failed dissolution therapy by alkalinisation of urine for radiolucent stones. A multi-detector NCCT examination of the abdomen and pelvis was performed in all patients. The stones were isolated and displayed in ‘Volume Rendering Technique’ using four-colour encoding.

**Results:**

Eight patients with failed dissolution therapy for radiolucent stones showed an outer layer of >500 Hounsfield units (HU) or a heterogeneous composition. A total of 42 patients with failed SWL had mean attenuations of <1000 HU on NCCT. Subsequent colour-coded stone mapping showed a dense core in all stones (>1000 HU) that failed to be clearly demonstrated by the mean HU alone.

**Conclusion:**

The initial use of a colour-coded density-gradients stone mapping reporting system for stone density on NCCT is useful for explaining failure of SWL or failure of dissolution therapy for radiolucent stones in selected cases.

**Abbreviations**: HU: Hounsfield units; MSD: mean stone density; NCCT: non-contrast computed tomography; PCNL: percutaneous nephrolithotomy; SWL: shockwave lithotripsy; VRT: Volume Rendering Technique

## Introduction

The last three decades has witnessed revolutionary development in the treatment of urinary stones, including shockwave lithotripsy (SWL) and percutaneous nephrolithotomy (PCNL), with different fragmentation techniques. The choice of the best treatment modality for each stone is very important to avoid unnecessary manoeuvres. This is especially important in choosing SWL as a treatment for renal or ureteric stones. It is well-known that a significant percentage of stones fail to fragment during SWL [1]. Unnecessary SWL has its side-effects, which could be avoided if we had better tools to predict stone fragmentation by SWL [2].

Non-contrast computed tomography (NCCT) is currently the ‘gold standard’ for stone imaging and prediction of stone fragility. Other imaging modalities, such as colour Doppler ultrasonography and ultrafast echo time MRI sequences have proven to be inferior to NCCT in the diagnosis of urinary stones and prediction of stone fragility [3]. Measurement of stone density in Hounsfield units (HU) using NCCT is the preferred way to predict stone fragmentation, using a cut-off value of 970 HU [3], 750 HU or <1000 HU as an indicator of stone fragmentation during SWL [4]. Nevertheless, there are still cases of failures of SWL and it seems that HU measurement alone is not enough for prediction of stone fragmentation. It has also been found that there is great variability between different brands and models of CT scanners in the assessment of attenuation values of different types of stones [5]. Uric acid stones show low attenuations (<600 HU) on NCCT, but not all stones with low attenuation respond to dissolution therapy [4,6].

The present study presents a novel clinically oriented system to report stone attenuation on NCCT using colour-coded density-gradients stone mapping and its clinical applications.

## Patients and methods

After approval of the local Ethics Committee, this study was performed prospectively on 50 patients with 63 stones (33 renal and 30 ureteric stones). Inclusion criteria included radio-opaque renal stones in the renal pelvis or upper calyx with a maximum diameter of ≤2 cm, radiolucent renal stones of any size, and upper ureteric stones with a maximum diameter ≤1.5 cm. All patients had to have a recent history of failed SWL or failed dissolution therapy by alkalinisation of urine for radiolucent stones. The failed SWL was performed using the Modularis Variostar lithotripter (Siemens, Erlangen, Germany) with patients in a supine position. Each patient received a maximum of three sessions. The therapy head of the electromagnetic lithotripter was positioned below the treatment table and conductive gel was applied. Every session started at the lower power with gradual escalation in steps every 100 shocks until the power was set to between 14 and 17 kV. The rate of shocks delivered was 60–90/min. The number of shockwaves was limited to 3000/session. Failed SWL was defined as the presence of a residual fragment of >0.7 cm in maximum diameter after three sessions of SWL. The failed dissolution therapy was performed using potassium sodium hydrogen citrate (6:6:3:5) granules at a dose of four measuring spoonful of granules (2.5 g) per day, with close monitoring of urinary pH to maintain a urine pH at ~6.8. It was given for 2 months.

**Figure 1.**
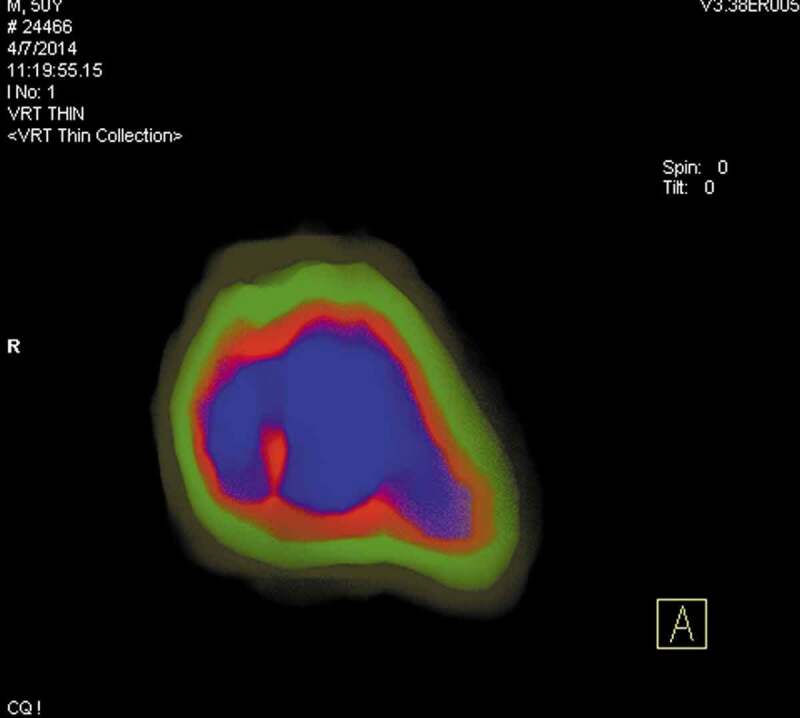
Colour-coded density-gradients stone mapping

Each patient underwent a plain X-ray of the abdomen and pelvis and multi-detector CT examination of the abdomen and pelvis with no oral or intravenous contrast administration (CT Stone protocol). CT examinations were performed by a single radiologist using 128-slice CT Siemens SOMATOM definition AS, with a collimation of 16 × 1.2 mm, voltage of 120 kV. Combined Applications to Reduce Exposure (CARE) Dose D was used in all cases to adjust the mAs according to the body build to decrease radiation exposure. Images were reconstructed and reviewed at a slice thickness of 1.2 mm with no gap, in both soft tissue and bone windows. Stones were isolated in CT and displayed in Volume Rendering Technique (VRT) using four-colour encoding as follows:

- **Blue** for 100% transparency for voxels >1250 HU attenuation (Figure 1).
- **Red** with 75% transparency for voxels between 1000 and 1250 HU attenuation.
- **Green** with 50% transparency for voxels between 500 and 1000 HU attenuation.
- **Yellow** with 25% transparency for voxels <500 HU attenuation.

Thin VRT images with 5-mm slice thickness were used to display small denser attenuations deep within the stone whenever needed. The colour images were displayed as a transverse section of the widest area of the stone and as a three-dimensional image. Stone density was assessed by two methods:

1. Mean stone density (MSD) was measured using bone windows on the magnified, axial NCCT image of the stone in the maximal diameter, where the elliptical region of interest incorporated the largest cross-sectional area of stone without including adjacent soft tissue.
2. MSD attenuation ratio was calculated by dividing the mean HU of each stone by its cross-sectional area, as measured in the axial CT scan.

## Results

The mean (SD) age of patients was 46 (12.3) years; there were 29 males and 21 females. The mean (SD) body mass index was 27.8 (5.4) kg/m^2^.

**Figure 2.**
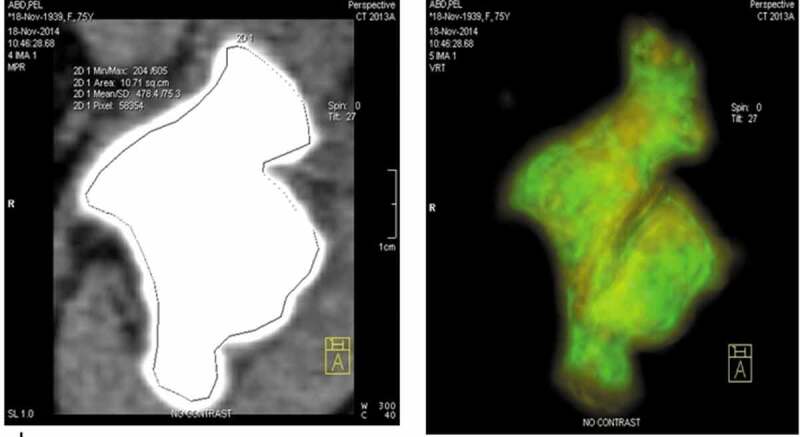
Colour-coded density-gradients stone mapping in a case of failed dissolution therapy showing heterogeneous composition of the stone

Eight patients had radiolucent renal stones (invisible on plain X-ray) and failed dissolution therapy by alkalinisation of their urine. The decision to use dissolution therapy was previously taken based on a stone density on CT of <500 HU. The MSD for these cases ranged from 480 to 570 HU, while the MSD attenuation ratio ranged from 416 to 514 HU. Colour-coded stone imaging showed an outer thin layer with a density of >500 HU or heterogeneous composition of the stone (Figure 2).

**Figure 3.**
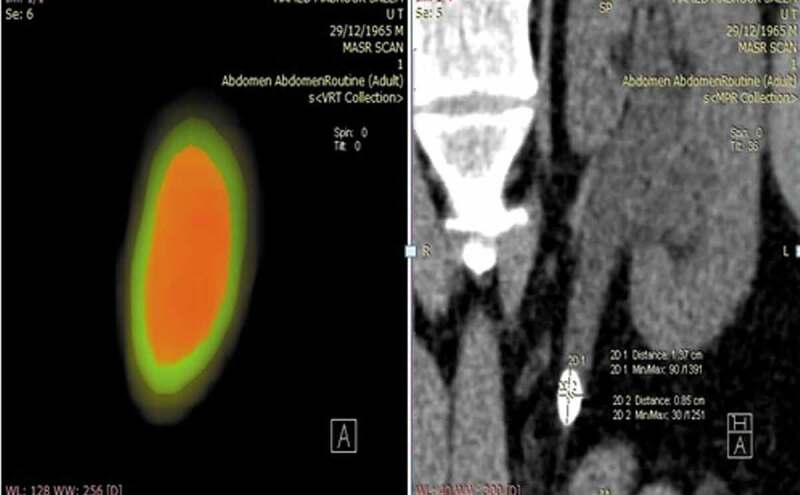
Failed SWL, note the hard core of the stone on performing stone mapping

In the 42 patients with recent history of failed SWL, all stones had a mean attenuation of <1000 HU on NCCT performed prior to SWL. The MSD for these ranged from 920 to 1340 HU, while the MSD ratio ranged from 830 to 1050 HU. Subsequent colour-coded stone mapping showed a dense core or a part of the stone with attenuation of >1000 HU (Figure 3), which was not clearly displayed by the mean HU alone. Of these 42 patients, 40 underwent flexible ureteroscopy and holmium laser lithotripsy, and the remaining two patients were treated with PCNL.

## Discussion

The NCCT stone protocol is considered the imaging technique of choice for diagnosis of urolithiasis and building clinical decision. Measurement of the mean attenuation and maximum HU is helpful to identify the possible stone composition to predict its fragility especially before SWL [4,6]. Realising the importance of stone volume in addition to HU attenuation as a predictor of stone fragmentation, other authors measured the stone attenuation ratio (mean attenuation number over the surface area of the stone at its maximal diameter) to give a better prediction of stone fragility [7]. However, these authors admitted setting 100 HU as a lower boundary of the stone attenuation value and they calculated the attenuation value measurements and stone volume from the voxels with an attenuation value of >100 HU, which may lead to overestimation of the stone size because of partial volume averaging of the edge voxels [7].

It seems that there are other factors than stone HU attenuation that affect stone fragility [8]. Early studies brought our attention to the effect of internal structure of the stone as a predictor parameter of stone fragility [9]. Thorough examination of the plain X-ray showed that there are four distinct radiological patterns of calcium oxalate stones depending on their relative content of calcium oxalate monohydrate or dehydrate and the pre-treatment recognition of these patterns may affect the selection of a therapeutic modality, but this is not applicable to all types of stones and has the great disadvantage of being a completely subjective method without standardisation [8,10,11].

The simultaneous use of both single- and dual-energy multi-detectors has been shown to predict renal stone composition and accordingly its fragility [12,13]. This dual-voltage CT is a new technology that can provide more data than single-voltage CT in genitourinary imaging [12]. It has been claimed that dual-voltage CT is able to differentiate most common stone types (struvite, cystine and calcium oxalate) when combined with single-voltage CT [13]. Other studies failed to show an advantage of dual-energy CT in identifying stone types when single-energy CT failed to do so [13]. This led to the limited implementation of dual-energy CT for stone identification, in addition to the fact that this technology is not widely available. Also, further software refinements are needed to improve the accuracy of dual-energy CT in stone identification, and more studies are necessary to assess the reproducibility and limitations of its images before it can be used on a wide scale [13]. Other sophisticated techniques, such as X-ray coherent scatter [14] or micro CT, can be utilised to study the internal structures of intact urinary stones, but these have the disadvantage of being desktop devices used only for *in vitro* studies [14].

An argument could arise saying that instead of performing colour-coded stone mapping, it is possible in radiolucent stones to measure density of the outer layer and in stones subjected to SWL, it is possible to measure the density of the core. If the outer layer measures >500 HU and if the core is >1000 HU, we can predict no/partial response to dissolution therapy and SWL. The answer to this argument is that colour-code stone mapping produced easily comprehensible images, which provided a clear view of the spatial structure of urinary stone in relation to its content with different attenuations and structures. In some radiolucent stones the cause of failed dissolution was the heterogeneous content of the stone and not just a dense outer layer. This was also found in some cases of failed SWL, where a part of the stone (not necessarily the core) was found by colour-coding to have an attenuation of >1000 HU.

A major limitation of the present study is the lack of a ‘gold standard’, e.g. the non-correlation between colour mapping and the results of stone analysis after stone extraction. Other limitations include the small number of cases studied and absence of a control group with successful SWL or dissolution therapy. Larger cohort studies are needed to study the clinical correlation between using a colour-coded density-gradients stone mapping reporting system for stone density and the success rate of non-invasive therapeutic modalities.

In conclusion, the initial use of a colour-coded density-gradients stone mapping reporting system for stone density on NCCT is useful for explaining failure of SWL or failure of dissolution therapy for radiolucent stones in selected cases.
